# Supplementary material for: What predicts response to sertraline for people with depression in primary care? a secondary data analysis of moderators in the PANDA trial
Source: PLoS One. 2024 May 9;19(5):e0300366. doi: 10.1371/journal.pone.0300366 (PMC11081306; doi:10.1371/journal.pone.0300366)
Supplement: Attachment — Submitted filename: renamed_c04c1.docx [file pone.0300366.s001.docx]

# COMMENTS – Manuscript PONE-D-23-29027

Title: “What predicts response to antidepressants for people with depression in primary care? An analysis of moderators in the PANDA trial”

**Important note: This review pertains only to ‘statistical aspects’ of the study and so ‘clinical aspects’ [like medical importance, relevance of the study, ‘clinical significance and implication(s)’ of the whole study, etc.] are to be evaluated [should be assessed] separately/independently. Further please note that any ‘statistical review’ is generally done under the assumption that (such) study specific methodological [as well as execution] issues are perfectly taken care of by the investigator(s). This review is not an exception to that and so does not cover clinical aspects {however, seldom comments are made only if those issues are intimately / scientifically related & intermingle with ‘statistical aspects’ of the study}. Agreed that ‘statistical methods’ are used as just tools here, however, they are vital part of methodology [and so should be given due importance]. I look at the manuscript in/with statistical view point, other reviewer(s) look(s) at it with different angle so that in totality the review is very comprehensive. However, there should be efforts from authors side to improve (may be by taking clues from reviewer’s comments). Therefore, please do not limit the revision only (with respect) to comments made here.**

**COMMENTS:** There are a few serious issues about which I have different opinion and such observations/concerns are given below:

Firstly, the ‘Title’ may mention that it is a secondary data analysis [nothing wrong in secondary data analysis, only it should be clear to potential readers, I guess]. This is (anyway) mentioned later on page 5 [We conducted secondary data analyses of PANDA trial [17] data to examine potential moderators of response to antidepressant treatment] anyway.

Secondly, one very basic/fundamental question: In the back drop of the fact that the original trial article [Lewis G, Duffy L, Ades A, Amos R, Araya R, Brabyn S, et al. The clinical effectiveness of sertraline in primary care and the role of depression severity and duration (PANDA): a pragmatic, double-blind, placebo-controlled randomised trial. The Lancet Psychiatry. 2019;6(11)] clearly concludes “the main benefits in the first 6 weeks of treatment with sertraline are on reduction of anxiety symptoms, such as worry and restlessness, rather than an improvement in depressive symptoms” and also says that “Our finding that sertraline was not effective for depressive symptoms at 6 weeks is inconsistent with previous studies”, I wonder if this database is expected to through light on problem/question in hand [“What predicts response to antidepressants for people with depression in primary care?”] for the very purpose of this small ‘paper exercise’ [[i.e., no design or execution or data collection]? In ‘Strengths and limitations’ section, though it is stated that “the PANDA trial was not designed to detect moderators of treatment effect and therefore is likely to be underpowered to estimate interaction effects robustly. Thus, the results of the present study should be interpreted with caution” however, one can not do away just by stating/adding such cautionary note, in my opinion.

Next, refer to ‘Methods-Outcome’ section where it is stated that “primary outcome for PANDA was the total score at 6 weeks on the Patient Health Questionnaire-9 (PHQ-9) and to maximise power this was treated as a continuous variable” which is totally “DISAGREED” because it is very erroneous. This is not the correct way to maximise/increasing/enhancing the power. Statement (on face value) that ‘treating any non-continuous variable as a continuous variable maximises (increase) the power’ is not only surprising but [such an erroneous statement] is not expected from present learned authors’ team. If you have something else in mind, please clarify. This is my first-hand {natural & immediate} reaction on the statement as it appears [& as I have understood it]. Please remember that this is a scientific/academic document and so all details should be clearly/correctly communicated (do not take readers’ for granted). In next section [Potential moderators] you said “In many cases the potential moderators were for the purposes of clarity of interpretation and presentation considered as categories; however, to maximise power many of the quantitative(?) measures were also analysed as continuous variables.” Is that correct? Surprising/wonderful.

Mind you that the PHQ‐9 (used here, though correctly, as a brief depression severity measure) is likely to yield data that are in ‘ordinal’ level of measurement [and not in ratio level of measurement for sure {as the score two times higher does not indicate presence of that parameter/phenomenon as double}]. Then application of suitable non-parametric (or distribution free) test(s) is/are indicated/advisable [even if distribution may be ‘Gaussian’ (also called ‘normal’)]. May check (level of measurement) for other scores (example: CIS-R depression severity score, social support score).

There is clear mention about ‘allocation’ {that Patients were randomly assigned (1:1) with a remote computer-generated code to sertraline or placebo, and were stratified by severity, duration, and site with random block length} in original trial article, but in present article (expected as this is an independent article and not one in series) there no such indication. More discussion on ‘sample size’ is also expected. Only saying that “The use of data from the PANDA trial enabled analysis of a sample size of 550 patients, which is larger than many other similar studies” is not at all sufficient, in my opinion.

As pointed out in ‘important note’ above “This review pertains only to ‘statistical aspects’ of the study and so ‘clinical aspects’ should be assessed separately/independently [one should carefully consider/look at the clinical implications of the study]. In my opinion, to make this article acceptable (which is quite possible and easy), a small amount of re-vision (re-drafting) may be needed. The respected ‘Editor’ may consider accepting/further processing as otherwise the draft is nicely worded. ‘Major revision’ is recommended, assuming that the respected editor would like to give chance to authors for improvement of the manuscript.
